# Current Landscape of Nutrition Within Prehabilitation Oncology Research: A Scoping Review

**DOI:** 10.3389/fnut.2021.644723

**Published:** 2021-04-09

**Authors:** Chelsia Gillis, Sarah J. Davies, Francesco Carli, Paul E. Wischmeyer, Stephen A. Wootton, Alan A. Jackson, Bernhard Riedel, Luise V. Marino, Denny Z. H. Levett, Malcolm A. West

**Affiliations:** ^1^Department of Anesthesia, McGill University, Montreal, QC, Canada; ^2^Department of Dietetics/Speech and Language Therapy, University Hospital Southampton NHS Foundation Trust, Southampton, United Kingdom; ^3^Duke Clinical Research Institute, Duke University School of Medicine, Durham, NC, United States; ^4^Faculty of Medicine, School of Human Development and Health, University of Southampton, Southampton, United Kingdom; ^5^National Institute of Health Research Cancer and Nutrition Collaboration, Southampton, United Kingdom; ^6^National Institute for Health Research Biomedical Research Centre, University Hospital Southampton National Health Service Foundation Trust, Southampton, United Kingdom; ^7^Department of Anaesthesia, Perioperative and Pain Medicine, Peter MacCallum Cancer Centre, Melbourne, VIC, Australia; ^8^Anaethesia, Pain and Perioperative Medicine Unit, The University of Melbourne, Melbourne, VIC, Australia; ^9^Centre for Integrated Critical Care Medicine and The Sir Peter MacCallum Department of Oncology, The University of Melbourne, Melbourne, VIC, Australia; ^10^Nutrition and Dietetics, Faculty of Health and Well Being, University of Winchester, Winchester, United Kingdom; ^11^Anaesthesia, Perioperative and Critical Care Research Group, National Institute for Health Research Biomedical Research Centre, University Hospital Southampton National Health Service Foundation Trust, University of Southampton, Southampton, United Kingdom; ^12^Faculty of Medicine, School of Cancer Sciences, University of Southampton, Southampton, United Kingdom

**Keywords:** surgical nutrition, oncological nutrition, pre-operative, pre-surgery, prehabilitation

## Abstract

**Background:** Prehabilitation aims to improve functional capacity prior to cancer treatment to achieve better psychosocial and clinical outcomes. Prehabilitation interventions vary considerably in design and delivery. In order to identify gaps in knowledge and facilitate the design of future studies, we undertook a scoping review of prehabilitation studies to map the range of work on prehabilitation being carried out in any cancer type and with a particular focus on diet or nutrition interventions.

**Objectives:** Firstly, to describe the type of prehabilitation programs currently being conducted. Secondly, to describe the extent to which prehabilitation studies involved aspects of nutrition, including assessment, interventions, implementation, and outcomes.

**Eligibility Criteria:** Any study of quantitative or qualitative design that employed a formal prehabilitation program before cancer treatment (“prehabilitation” listed in keywords, title, or abstract).

**Sources of Evidence:** Search was conducted in July 2020 using MEDLINE, PubMed, EMBASE, EMCARE, CINAHL, and AMED.

**Charting Methods:** Quantitative data were reported as frequencies. Qualitative nutrition data were charted using a framework analysis that reflects the Nutrition Care Process Model: assessment, intervention, and monitoring/evaluation of the nutrition intervention.

**Results:** Five hundred fifty unique articles were identified: 110 studies met inclusion criteria of a formal prehabilitation study in oncology. prehabilitation studies were mostly cohort studies (41%) or randomized-controlled trials (38%) of multimodal (49%), or exercise-only (44%) interventions that were applied before surgery (94%). Nutrition assessment was inconsistently applied across these studies, and often conducted without validated tools (46%). Of the 110 studies, 37 (34%) included a nutrition treatment component. Half of these studies provided the goal for the nutrition component of their prehabilitation program; of these goals, less than half referenced accepted nutrition guidelines in surgery or oncology. Nutrition interventions largely consisted of counseling with dietary supplementation. The nutrition intervention was indiscernible in 24% of studies. Two-thirds of studies did not monitor the nutrition intervention nor evaluate nutrition outcomes.

**Conclusion:** Prehabilitation literature lacks standardized and validated nutritional assessment, is frequently conducted without evidence-based nutrition interventions, and is typically implemented without monitoring the nutrition intervention or evaluating the intervention's contribution to outcomes. We suggest that the development of a core outcome set could improve the quality of the studies, enable pooling of evidence, and address some of the research gaps identified.

## Background

Prehabilitation interventions can be applied prior to oncological treatments, including surgery, to fortify functional reserve and enhance functional capacity to prepare patients to weather the imminent physiological and psychological stresses of treatment (1). Preoperative functional capacity is predictive of postsurgical outcomes, such as morbidity in colorectal surgery (2, 3). As an example, frail patients who cannot attain a 400-m 6-min walking distance before surgery suffer three times as many postsurgical complications as those who can walk this distance (2). In the same way, there is an extensive body of evidence that those who are undernourished, as marked by a history of weight loss and symptoms indicative of poor nutritional state, have greater surgical morbidity and mortality (4). Several prospective studies have identified that unimodal (e.g., exercise-only interventions) and multimodal (e.g., exercise interventions with nutrition optimization and/or psychological intervention) prehabilitation programs can be carried out successfully in the period before surgery to improve preoperative functional capacity (5–8).

The findings of available systematic reviews of prehabilitation, however, are somewhat inconsistent regarding effectiveness of the intervention on clinical outcomes such as postoperative complications (9, 10). These seeming contradictions are in part related to the heterogeneity of study populations, study designs, and study interventions that often cannot be melded together into one message for prehabilitation (11). Undernutrition, for instance, leads to adaptive mechanisms that tend to reduce energy expenditure in part by reducing physical activity and basal metabolism with conservation of reserves (12). As a result, malnourished patients participating in exercise-only prehabilitation might not be able to engage with or adapt to exercise and improve their functional capacity prior to surgery as well as those who are better nourished (2). The inconsistent findings of these reviews may also be attributed to the scarcity of process measures/implementation outcomes reported in the prehabilitation literature. Synthesizing and reporting data on the effectiveness of an intervention *only* limits conclusions: success or failure of any intervention is a combination of treatment effectiveness (in terms of both improved functional endpoints, and the impact on clinical outcomes, e.g., reduced postoperative complications) together with its implementation factors (13). Few, if any, reviews of prehabilitation have reported implementation factors that might influence the effectiveness of the program.

While systematic reviews summarize and assess the quality of the collective evidence of a given topic, scoping reviews determine the coverage of a body of literature on a specific topic to identify the available evidence, to examine how research in the field was conducted, and to identify and assess knowledge gaps (14). We conducted a scoping review to determine *what* and *how* interventions have been incorporated as part of prehabilitation in the oncology setting. That is, we sought to identify the type of interventions currently being conducted within prehabilitation programs, the patient populations being studied, and the study designs that have been used in research specifically labeled as “prehabilitation” (i.e., “what”). Additionally, given the relationship between nutrition and functional capacity, we sought to determine the extent to which prehabilitation studies involved nutrition, including assessment, interventions, implementation, and outcomes (i.e., “how”). We aimed to identify any research limitations or omissions that could usefully inform future research design, conduct and interpretation, or that could help improve the coherence and delivery of the nutritional aspects of prehabilitation in clinical practice.

## Methods

We performed a scoping review of the literature based on the framework outlined by Arksey and O'Malley (15), recommendations of Levac et al. (16), and in accordance with the Preferred Reporting Items for Systematic reviews and Meta-Analysis extension for Scoping Reviews (PRISMA-ScR). The review included the following five key phases: (1) identifying the research question, (2) identifying relevant studies, (3) study selection, (4) charting the data, and (5) collating, summarizing, and reporting the results. A project team consisting of health researchers, physicians, dietitians, an epidemiologist, and perioperative clinic managers was established to develop the research question and oversee the study.

### Identifying the Research Question

The overarching goal of this scoping review was to provide an overview of current prehabilitation practices in oncology, to identify the extent to which prehabilitation programs included nutrition, and to generate recommendations for future studies based on identified gaps. Our research questions were as follows:

1. What are the study, patient, and intervention characteristics of published prehabilitation studies?
2. How many prehabilitation studies were conducted with a nutrition treatment component?
3. What are the specific (i) nutrition assessments, (ii) interventions, (iii) process measures (monitoring and evaluation), and (iv) nutrition outcomes associated with the prehabilitation studies that included a nutrition treatment component?

### Identifying Relevant Studies

Given that our goal was to map current research practices in oncology-related prehabilitation, we focused our scoping review to studies of interventions applied prior to oncology treatment that were identified as either unimodal or multimodal prehabilitation; that is, published work, including protocols, that contained the term “prehabilitation” in the title, abstract, or keywords. We did not set a time limit to the search to ensure as much evidence as possible was captured.

We used broad search terms that encompassed prehab^*^ or pre-hab^*^ or pre-rehab^*^ AND cancer^*^ or oncolog^*^ or malignan^*^. The final search was conducted in July 2020 using MEDLINE, PubMed, EMBASE, EMCARE, CINAHL, and AMED. Hand searching the reference lists of key papers, including all identified systematic reviews and meta-analyses of prehabilitation, were also conducted.

### Study Selection

Two reviewers (CG and SD) independently reviewed titles and abstracts for inclusion. Articles were considered for full-text review if inclusion criteria were met: (1) a quantitative or qualitative study of a “prehabilitation” program; and (2) included adult patients (age >18 years) with cancer (or where the majority of participants reported in the study had cancer), treated with surgery or other oncological therapies. Studies were excluded if they were narrative reviews, editorials, commentaries, conference abstracts, or were published in a language other than English or French. Selected articles for full-text review were then independently reviewed by the two reviewers. Disagreements were addressed by discussion and consensus.

### Charting the Data

The data extraction template (Microsoft 2010, Redmond, WA) was developed in consultation with the project team and included study design, cancer type, specification of the prehabilitation program, primary outcome measure, and whether nutrition was part of the formal prehabilitation program by including the use of nutritional screening/assessment or nutrition treatment. Of the studies identified as having a nutrition intervention component, quantitative and qualitative data were collected on: (1) method of nutritional assessment, (2) validated nutrition screening or assessment tool, (3) goal of the nutrition intervention including the reference standard or accepted nutritional guideline, (4) characteristics of the nutrition intervention, (5) evaluation and monitoring of the intervention, and (6) nutrition outcomes. Two researchers (CG and SD) independently extracted data for the first 10 studies to refine the data form and ensure consistent data extraction that adequately reflected the research question.

### Collating and Summarizing Results

Quantitative data were analyzed using descriptive statistics (frequencies). Qualitative data were charted using a framework analysis that reflects the Nutrition Care Process Model: assessment, intervention, and monitoring/evaluation of the nutrition intervention (17). The study team were consulted in the interpretation of the findings, identifying research gaps and creating suggestions for future research.

## Results

### Search Results

**Figure 1:**
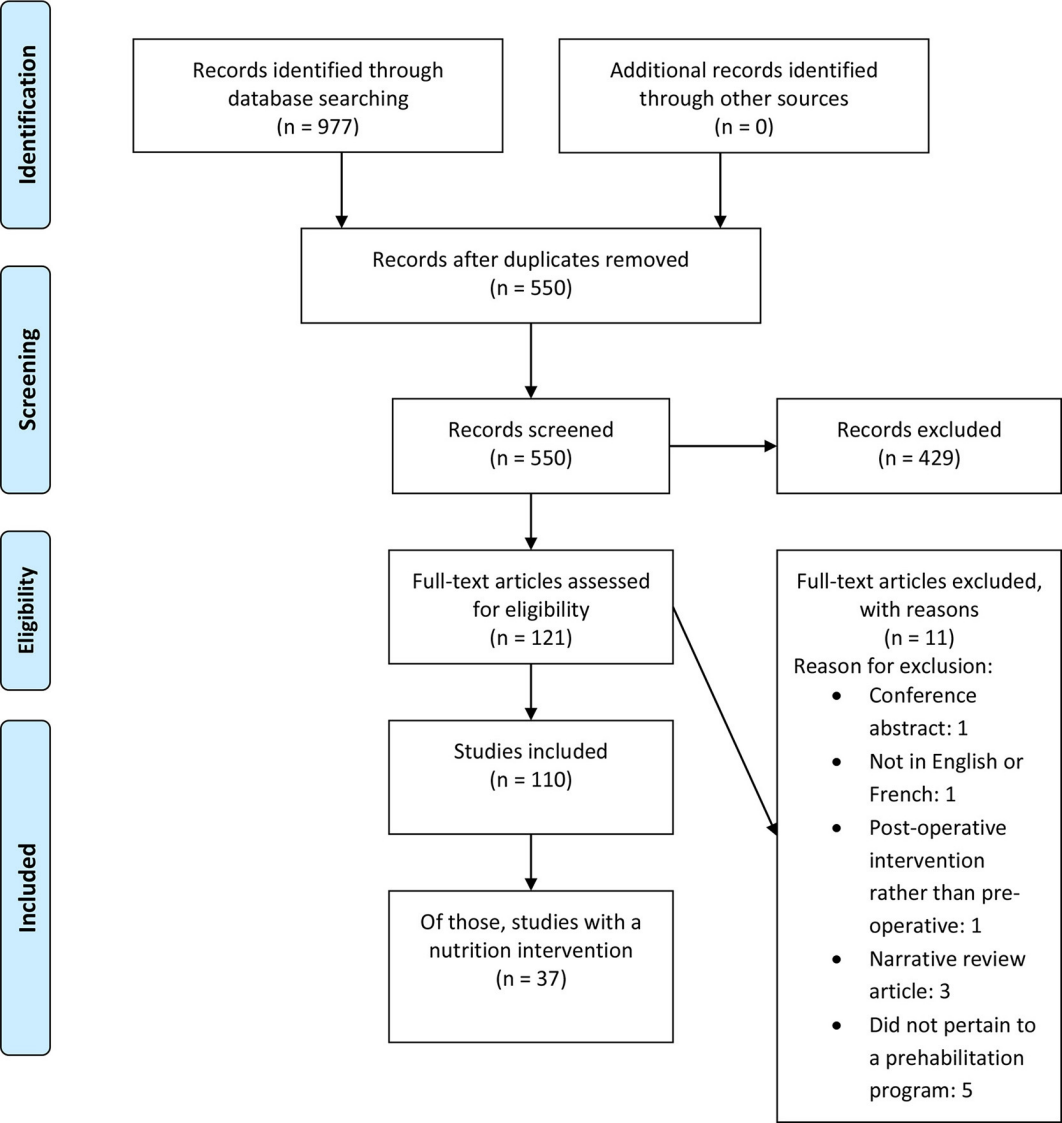
Flow chart of search results.

Our search identified 550 unique articles (Figure 1). After abstract screening, 121 articles were suitable for full-text review. Hand searching did not produce any further unique articles. Eleven articles were subsequently excluded because of language (*n* = 1), a narrative review (*n* = 3), a conference abstract (*n* = 1), no preoperative intervention (*n* = 1), or did not pertain to a prehabilitation program (*n* = 5). One-hundred and ten studies were included in the final review, of these, 34% (*n* = 37) included a nutrition intervention component.

### All Prehabilitation Studies

Table 1 describes the findings for all of the prehabilitation studies. These studies were published between 2012 and 2020. Of these 110 studies, 56% (*n* = 61) were identified as primary research studies; 57% of the prehabilitation studies arose from Europe (*n* = 63) and 21% from Canada (*n* = 23). The primary studies were largely conducted as cohort designs (*n* = 25; 41%) and randomized controlled trials (RCTs) (*n* = 23; 38%). Systematic reviews, meta-analyses, and pooled analyses comprised 23% (*n* = 25) of the prehabilitation literature. Functional (*n* = 40; 36%) and clinical (*n* = 25; 23%) measures were the most frequently reported primary outcomes.

**Table 1:** Patient, study, and intervention characteristics of all prehabilitation studies.

**Characteristic**	**Number of studies (*n* = 110)**	**Percentage (%)**
**A. ALL PREHABILITATION STUDIES**		
***Study characteristics***		
**Country**		
Europe	63	57.3
Canada	23	20.9
United States	15	13.6
Asia	4	3.6
Australia	5	4.6
**Published studies**		
Primary studies	61	55.5
Secondary analysis	8	7.3
Systematic review	16	14.5
Meta/pooled analysis	9	8.2
Protocol	13	11.8
Implementation study/description of prehabilitation implementation	3	2.7
**Study design of primary studies**		
Randomized controlled trial	23	37.7
Cohort study	25	40.9
Case report	4	6.6
Pilot	9	14.8
**Primary outcome**		
Functional	40	36.4
Clinical	25	22.7
Patient reported	9	8.2
Nutrition outcome	1	0.9
Feasibility	17	15.5
Mixed primary outcomes	3	2.7
Not applicable/ not specified	15	13.6
**Indication for prehabilitation**		
Surgery	103	93.6
Definitive oncological treatment	7	6.4
***Patient characteristics***		
**Cancer type**		
Colorectal	35	31.8
Lung	9	8.2
Pancreatic	4	3.6
Bladder	4	3.6
Gastric	1	0.9
Esophageal	4	3.6
Breast	4	3.6
Prostate	7	6.4
Hematological	4	3.6
Head and neck	2	1.8
Brain	1	0.9
Gynecological	1	0.9
Mixed cancer cohort	33	30.0
Not specified	1	0.9
***Intervention characteristics***		
**Prehabilitation intervention**		
Exercise only	48	43.6
Nutrition only	2	1.8
Psychology only	1	0.9
Function only	5	4.6
Multimodal	54	49.1
**B. PREHABILITATION STUDIES WITH NUTRITION SCREENING OR ASSESSMENT**		
**Was a nutrition screen or assessment performed?**		
Yes	33	30.0
No	48	43.6
Not specified	12	10.9
Not applicable^*^	17	15.5
**Was at least one validated screening or assessment tool used?**		
Yes	17	51.5
No	15	45.5
Not specified/ enough information available	1	3.0
**Was the screening or assessment performed by a registered dietitian?**		
Yes	13	39.4
No	5	15.2
Not specified	15	45.4

* *Not applicable refers to any study that did not collect primary data*.

Most of the prehabilitation literature described multimodal (*n* = 54, 49%) or exercise-only prehabilitation (*n* = 48, 44%); two studies reported interventions that were exclusively nutrition related (2%) while one study reported an intervention that was exclusively psychological (1%). We identified that surgical prehabilitation made up 94% of the literature, with the rest related to definitive non-surgical oncological treatments. The patient populations studied most were colorectal cancer (*n* = 35; 32%) and mixed cancer types (*n* = 33; 30%).

Screening or assessment for malnutrition was conducted in one-third of prehabilitation studies (*n* = 33); approximately half of these studies used a validated tool (*n* = 17) and 39% of these studies (*n* = 13) employed a registered dietitian to conduct the screening or assessment. The person who conducted the screening/assessment was not specified in 45% of these studies.

### Prehabilitation Studies With a Nutrition Treatment Component

Table 2 and Supplementary Table 1 describe the quantitative and qualitative findings of the prehabilitation studies with a nutrition treatment component. Only 37 of the 110 studies of prehabilitation had a nutrition treatment component. The study designs were as follows: 27% (*n* = 10) were protocols (18–27), 14% (*n* = 5) were pilot studies (8, 28–31), 5% (*n* = 2) were descriptions of prehabilitation programs (32, 33), 3% (*n* = 1) were case reports (34), 3% (*n* = 1) were feasibility studies (35), and 3% were qualitative studies (36). Of these 37 studies, 30% (*n* = 11) were cohort studies (37–47) and 16% (*n* = 6) were RCTs (48–53).

**Table 2:** Study and intervention characteristics of prehabilitation studies with a nutrition component.

	**Number of studies (*n* = 37)**	**Percentage (%)**
**STUDY CHARACTERISTICS**		
**Study design of primary studies**		
Randomized controlled trial	6	16.2
Cohort study	11	29.7
Case report	1	2.7
Pilot	5	13.5
Feasibility	1	2.7
Protocol	10	27.0
Implementation study/description of prehabilitation implementation	2	5.4
Qualitative study	1	2.7
**Indication for prehabilitation**		
Surgery	37	100.0
Definitive oncological treatment	0	0
**INTERVENTION CHARACTERISTICS**		
**Was a nutrition screen or assessment performed?**		
Yes	29	78.4
No	8	21.6
**Was an explicit goal stated for the intervention?**		
Yes	21	56.8
No	16	43.2
**If a goal was stated, was this referenced?**		
Surgery or oncology guideline	9	42.9
Expert consensus	2	9.5
Another study referenced	3	14.3
No reference provided	7	33.3
**What was the nutrition intervention?**		
Supplementation only	3	8.1
Counseling only	3	8.1
Counseling (generalized or personalized) in addition to supplementation	19	51.3
Leaflet	2	5.4
Ingredients provided	1	2.7
Not enough information provided	9	24.3
**If supplementation was provided, what was the type of supplementation**		
Protein supplements	11	50.0
Protein supplements in addition to vitamin and/or mineral supplementation	3	13.6
High energy oral nutritional supplements	1	4.6
Immunonutrition	1	4.6
Leucine	1	4.6
Not specified	5	22.7
**Was the nutrition intervention monitored or evaluated?**		
Yes	11	29.7
No/not specified	26	70.3
**Were any nutrition-related outcomes reported?**		
Yes	16	43.2
No	21	56.8

### Nutritional Assessment Within Prehabilitation

Seventy-eight percent (*n* = 29) of the 37 identified studies included a statement regarding the conduct of nutritional assessment [*n* = 8 studies did not include a nutritional assessment statement (20, 26, 32, 36, 39, 43, 45, 47)]; however, the application of assessment was inconsistent across studies. Each study used a different method for nutritional assessment, with most studies using a combination of various nutritional assessment tools, parameters, and indicators. The most commonly used tools to screen or assess for malnutrition were Subjective Global Assessment/Patient-Generated-Subjective Global Assessment (8, 27, 31, 35, 51), Nutrition Risk Screening-2002 (8, 19, 51, 52), Mini Nutritional Assessment (23, 28, 40, 41), Simplified Nutritional Appetite Questionnaire (23, 37, 41), and Malnutrition Universal Screening Tool (22, 46). The most common nutritional parameters were pre-albumin or albumin (18, 19, 23, 34, 38, 41, 46), which were reported by 19% (*n* = 7) of studies as a nutritional parameter [although, it is not considered to robustly reflect nutritional status in patients with cancer (54)], and 27% (*n* = 10) reported use of food records or recalls (8, 18, 27, 34, 35, 48–51, 53). Forty-three percent (*n* = 16) of studies included nutritional indicators, such as weight, body mass index (BMI), or body composition as an element of the assessment (18, 19, 23, 27–30, 33, 35, 38, 40, 41, 44, 46, 50, 53). Body composition analysis included computed tomography (CT) (18), bioimpedance (19), and skinfold assessments (24, 27, 35).

Eight percent (*n* = 3) of studies stated that an assessment was conducted without providing details of the method or tool used (21, 25, 42). As examples, “Complete nutritional assessment undertaken by a registered dietitian” (42) and “A nutritionist performed a medical examination running appropriate biological tests to evaluate the nutritional status” (25). Another study provided only vague details of the nutritional parameters used—“the dietitian assessed nutritional status using … and blood vitamin B [the B-vitamin assessed was not specified]” (41). In most cases, the cut-points or criteria for nutritional risk or diagnosis of a nutrition problem requiring treatment (e.g., malnutrition) were not specified. Only 16% (*n* = 6) of studies specified their diagnostic criteria rather than cut-points (22, 23, 28, 40, 44, 46).

### Nutrition Interventions Within Prehabilitation

Eleven percent (*n* = 4) of studies specified that a nutrition intervention was provided to patients “in need” without defining the mechanism for identifying these patients (18, 20, 32, 47). As an example, “Usual care for all participants included review by specialist dietitians if they were struggling nutritionally (20).” Little more than half (*n* = 21) of the prehabilitation studies with a nutrition treatment component specified a goal for the nutrition intervention; of these, 67% (*n* = 14) referenced the stated goals and only 43% (*n* = 9) used a reference standard or accepted guideline, including European Society for Clinical Nutrition and Metabolism (ESPEN) guidelines (8, 21, 25, 35, 48–51, 53). Most goals were related to meeting estimated protein needs (8, 22, 25, 27, 28, 31, 35, 37, 48, 51, 53) or meeting estimated energy and protein needs (19, 21, 23, 39, 41, 49, 50). Protein needs were estimated at 1.2–2.0 g/kg/day and energy needs were estimated using 25–30 kcal/kg/day, indirect calorimetry, Harris Benedict equation, or WHO formula. Other stated nutrition goals included optimizing nutritional status (30), protein supplementation (32), and caloric and protein supplementation (18). Fifty-one percent (*n* = 19) of the interventions applied to meet these goals included a combination of both nutrition counseling (personalized or generalized) and supplementation (8, 18, 19, 22, 23, 25, 27, 31, 34, 35, 39, 41, 42, 48–53). Eight percent (*n* = 3) of studies used counseling alone (30, 44, 45), 5% (*n* = 2) used a leaflet (26, 36), and 8% (*n* = 3) used supplementation alone (32, 38, 46). Of the studies that used a nutrition supplement, “protein supplements” or a combination of vitamin/mineral supplements with protein supplements (8, 22, 25, 27, 31, 32, 34, 35, 38, 41, 48–51, 53) were used most often. Other supplements included high-energy oral nutrition supplements (19) and immunonutrition (46). Whey protein supplements (8, 22, 27, 31, 48–51, 53) were among the most prevalent of the protein-only supplements used in prehabilitation studies. Twenty-three percent (*n* = 5) of studies reported use of a supplement but did not provide any detail on the type of supplement used (18, 23, 39, 42, 52).

Many interventions appeared to be “personalized” to meet individual patient needs (8, 18, 19, 22, 24, 25, 32, 34, 39, 53). For some of the studies, it was clear that the nutrition assessment directed the nutrition care plan, including the need for specialized nutrition support (20, 40, 46), provision of a supplement or the supplemental dose (19, 23, 41, 49–51, 53), need for weight loss/gain (8, 27, 42, 53), or provided dietary advice based on food recalls, dietary patterns, and nutrition-impact symptoms (8, 22, 30, 31, 39, 51, 53). It was unclear how the nutritional assessment influenced the treatment plan in the remaining studies. Standardized instructions revolved around consuming protein supplements or snacks post-exercise (25, 27, 31, 35, 39, 45, 48–51, 53), increasing dietary protein intake (22, 27, 28, 34, 36, 50–52) and tips on consuming balanced meals (22, 44, 48, 53). Twenty-four percent (*n* = 9) of studies did not provide enough information for us to discern the specific nutrition intervention (20, 21, 24, 29, 33, 37, 40, 43, 47). Examples include, “aimed to incorporate nutrition support (33),” “appropriate supplementation (18),” or leaflets or seminars that “included nutrition (29, 43).”

### Monitoring and Evaluation of Nutrition Impact Within Prehabilitation

Finally, a third (*n* = 11) of studies monitored adherence to the nutrition intervention (8, 19, 22, 25, 28, 30, 35, 45, 49, 52, 53). Self-reported adherence using logbooks/dairies (8, 19, 50, 52, 53) and a mobile app (22) were reported. Twenty-four percent (*n* = 9) of studies monitored adherence and provided ongoing support through telephone calls (8, 19, 24, 28, 35, 45, 49, 50, 53). However, tailoring of the nutrition intervention based on a follow-up appointment or telephone call was reported in only 8% (*n* = 3) of studies (24, 25, 50). An objective evaluation of whether the nutrition prescription was meeting patient needs preoperatively was reported in only one study where weight was measured (30). Yet, 43% (*n* = 16) of the studies reported some form of nutrition outcome, such as weight (18, 24, 29, 30, 33, 35, 38, 44, 51), food records or questionnaire (18, 21, 27, 44), nutrition screening or assessment tools (19, 27, 35), body composition (8, 18–22, 24, 29, 51), and handgrip strength (8, 20, 24, 33, 35). Although food recalls/records were stated to be used in several studies, only one study reported intake data (fiber and fat) (44). Of note, only 5% (*n* = 2) of studies examined outcomes by sex (38, 51).

## Discussion

We conducted a scoping review to map the formal prehabilitation literature and identify opportunities to improve future research with particular emphasis on nutritional support. Currently, much of the available prehabilitation evidence, which could be used to inform practice and policy, is in the form of cohort studies. The majority of prehabilitation studies were conducted as multimodal or exercise-only studies and were applied before surgery. Only one-third of these studies included a dietary/nutrition treatment component. Nutrition assessment was inconsistently applied across these studies. In many studies, it was unclear how the nutrition assessment was used to identify nutrition problems or influence the treatment plan. Nearly one-quarter of these studies stated a nutrition intervention was applied without describing the intervention. Approximately half of the studies reported a nutrition treatment goal; yet, of those studies that reported a goal, one-third were not referenced at all and less than half referenced accepted nutrition guidelines in surgery or oncology. Finally, approximately two-thirds of studies did not monitor the nutrition intervention or evaluate nutrition outcomes.

This review identified several important research gaps. Firstly, two-thirds of the published literature on prehabilitation did not include nutrition risk screening or malnutrition assessment. Given that nutritional status can exert a modifying effect on nutritional (55), clinical (56, 57), and functional (58) outcomes, a failure to examine treatment effects at different levels of nutritional status limits research conclusions and clinical decision making (59–61). Effect modification is considered a natural phenomenon that should be reported and described; therefore, pooling of data should only be considered when the effect of treatment is identified to be homogenous across the strata of a potential modifying variable (e.g., nutritional status) (62). Considering a single treatment effect for prehabilitation on the impact of outcomes, independent of nutritional status, could result in a finding of a null effect (if subgroups respond to treatment in opposing ways), an overestimated, or an underestimated effect of prehabilitation treatment depending on the prevalence of malnutrition in the sample. Similarly, many studies were conducted in mixed cancer types, yet the treatment effect for prehabilitation might differ based on cancer status. While small sample sizes often preclude modification analysis, a failure to investigate heterogeneous effects could be a contributing factor to the conflicting, contradictory reports of the effect of prehabilitation on outcomes.

Overall, nutritional screening and assessment across published prehabilitation studies was heterogeneous and often completed without validated tools. Informal assessments, including clinical parameters and subjective measures result in under recognition of malnutrition (63). Valid nutritional assessment is required to identify malnutrition and any other nutrition-related problems that contribute to adverse outcomes. This finding has three important implications for prehabilitation research: (1) using non-validated tools to identify malnutrition produces findings that are subject to misclassification bias; (2) using a variety of tools to identify malnourished patients limits cross-study comparisons and synthesis of findings for meta-analysis; and (3) even validated tools cannot diagnose malnutrition with 100% sensitivity and specificity, so it is unlikely that the studies employing non-validated tools identified all the nutritionally compromised patients. The latter point is particularly problematic given that the primary outcome for most prehabilitation trials was identified to be functional and/or clinical. Malnourished patients have lower functional capacity (58, 64) and a reduced capacity to gain function through exercise alone (without first correcting malnutrition, which, for malnourished patients, could be the underlying etiology for the compromised function (58, 65, 66). A failure to correctly identify malnutrition for treatment has the potential to produce misleading findings for the effect of prehabilitation.

Of the published prehabilitation studies with a nutrition treatment component, approximately two-thirds of these studies did not monitor or evaluate the nutrition intervention. According to Proctor et al. (13), when an intervention fails to deliver, it is critical that we are able to attribute failure to either the intervention itself, the factors associated with its implementation, or a combination of the two. Inferring success or failure of the prehabilitation program using only functional and clinical endpoints is problematic as it is impossible to discern where the success or failure lies (13). As an example, we identified that 41% of nutrition prehabilitation interventions supplemented protein. Yet, it is difficult to discern whether positive or negative findings can be attributed to this intervention, or to another component of the multimodal prehabilitation, given implementation was poorly documented. If we have failed to monitor whether the nutrition prescription met patient needs (e.g., the intervention was acceptable to the patient, it was feasible to meet estimated therapeutic targets with the given intervention), assess implementation outcomes (e.g., fidelity of the intervention against protocol or patient adherence to the prescribed intervention), or evaluate nutrition outcomes (e.g., weight stabilization for malnourished patients), we cannot conclude with confidence that the intervention itself was (un)successful. Studies that do not monitor the nutrition prescription and evaluate the outcomes, do not contribute to our collective understanding of which interventions work best, how do they work, and for whom do they work best.

Finally, almost half of the published prehabilitation studies with a nutrition treatment component did not report the goal of the nutrition intervention. Several accepted standards exist to form the basis of nutrition goals in surgery (4) or oncology (67, 68) care. This finding has two major implications for prehabilitation research. First, when the goal of an intervention is unknown, critical appraisal of the study design and study's finding is difficult. Second, it is expected that evidence-based interventions that represent accepted standards are most likely to meet patient needs consistently. Treating patients without taking cognizance of and seeking to achieve these standards increases the risk of inadequate nutritional care with the associated inferior outcomes, again, potentially contributing to conflicting findings for multimodal or nutrition prehabilitation.

In order to effectively address the research gaps identified, we recommend that a core outcome set (COS) be developed and adopted for prehabilitation studies. A COS is a standardized set of outcomes to be reported by all trials within a research field (69). Additional outcomes may be reported at the discretion of the researcher, but a minimum standardized set of outcomes would be reported, permitting cross-study comparisons and enabling data synthesis for systematic reviews or meta-analyses that inform clinical practice (70). This need is illustrated by our identification that 23% of the formal prehabilitation literature constitutes systematic reviews and meta-analyses, and many of these reviews were found to be inconclusive, citing heterogeneity as the rationale. Clearly, addressing the extent of heterogeneity would enhance data synthesis and should be seen as a priority for prehabilitation research. For nutrition, the development of a COS that includes standards for nutritional assessment, a requirement to state the goal of the intervention in relation to an appropriate reference standard, along with a standard set of measurements to monitor and evaluate the intervention, could greatly advance the literature.

We would like to acknowledge a few limitations. First, we did not register this trial; although, this is not a prerequisite for scoping reviews. Second, this review was limited to prehabilitation interventions for patients with cancer. As a result, our findings should not be generalized to all prehabilitation research. Third, our search was limited to six databases and languages of English and French; these criteria may have biased our findings. Finally, we limited our review to formal prehabilitation studies (articles with the term prehabilitation in the title, abstract or keywords); this strategy may have introduced misclassification bias. That said, there is no accepted definition of prehabilitation, and our goal was to map the range of studies currently being conducted as a form of “prehabilitation.” We also acknowledge the large body of evidence of nutritional-only interventions such as preoperative nutritional support that have been reported previously that would not be included using our search strategy focusing on prehabilitation.

## Conclusion

The prehabilitation literature is lacking standardized and validated nutritional assessment, is frequently conducted without employing evidence-based nutrition interventions, and is typically conducted without monitoring the nutrition intervention or evaluating the intervention's contribution to outcomes. In order to advance our understanding of prehabilitation, the nutrition component of prehabilitation interventions should be based on validated tools of assessment, accepted standards, monitored, and evaluated. We suggest that the development, adoption, and application of a core outcome set would be a first step in addressing the research gaps identified and result in studies that are more likely to inform clinical practice and improve patient outcomes.

## Data Availability Statement

The original contributions presented in the study are included in the article/Supplementary Material, further inquiries can be directed to the corresponding author/s.

## Author Contributions

CG, SD, and MW designed the research. CG and SD carried out the data collection. All authors edited, read, and approved the final manuscript.

## Conflict of Interest

The authors declare that the research was conducted in the absence of any commercial or financial relationships that could be construed as a potential conflict of interest.
