# Supplementary material for: Current Landscape of Nutrition Within Prehabilitation Oncology Research: A Scoping Review
Source: Front Nutr. 2021 Apr 9;8:644723. doi: 10.3389/fnut.2021.644723 (PMC8062858; doi:10.3389/fnut.2021.644723)
Supplement: Supplementary file 1 [file Table_1.DOCX]

**Table 1: Qualitative description of formal prehabilitation studies with a nutrition component**

| **Author, year of publication and study design** | **Cancer type and sample size** | **Nutrition screening and assessment** | **Nutrition intervention** | **Monitoring of intervention** | **Nutrition outcomes** |
| --- | --- | --- | --- | --- | --- |
| Sell, 2020 (32)  Description of a prehabilitation program | *Cancer type*: Not specified  *Sample size*: Not applicable | No nutritional screening or assessment detailed | *Goal:* Patients consume supplemental protein intake of 30g per day four times per week in addition to their normal dietary intake  *Intervention:* Specific protein supplement (not specified) determined on an individual basis by the treating physician or licensed dietitian/nutritionist  *Reference for goal:* Minnella EM, Awasthi R, Loiselle SE, et al. Effect of Exercise and Nutrition Prehabilitation on Functional Capacity in Esophagogastric Cancer Surgery: A Randomized Clinical Trial. JAMA Surg. 2018;153(12):1081-1089 | Not specified | No nutrition outcomes measured |
| Tully, 2020 (18)  Protocol for RCT | *Cancer type*: Mixed (Esophageal and gastric)  *Sample size*: Aims to recruit n=62 | All patients will have a dietitian assessment at time of diagnosis. Nutritional status will be assessed using Glasgow Prognostic Score, Foodbook-24 (a web-based 24-hour dietary recall and food frequency questionnaire) and sarcopenia assessment will calculate skeletal muscle mass from diagnostic CT and restaging CT after neoadjuvant treatment | *Goal:* Ensure sufficient calorie and protein supplementation  *Intervention:* An individualized dietary plan with appropriate supplementation (not specified). Pre- and peri-operative feeding adjuncts (percutaneous enteral feeding or total parenteral nutrition) will be recorded on an individual basis  *Reference:* Not specified | Not specified | *Outcomes measured:* BMI, Glasgow prognostic score, Foodbook-24 (not specified what information will be drawn from these) and sarcopenia assessment  *Timepoint:* 4 weeks and 10 weeks postoperatively |
| Ploussard, 2020 (42)  Cohort study | *Cancer type*: Prostate  *Sample size*: n=194 | ‘Complete’ nutritional assessment undertaken by a registered dietitian | *Goal:* Not stated  *Intervention:* Dietitian intervention included a complete assessment with general nutritional advice. Underweight patients were given oral nutrition support with supplements (not specified) one week before surgery. Overweight patients were given dietetic counselling for weight loss.  *Reference:* Not specified | Not specified | No nutrition outcomes measured |
| Bausys, 2020 (19)  Protocol for a multi-centre RCT | *Cancer type*: Gastric  *Sample size*: Aims to recruit n=128 | Nutritional status measured at baseline and preoperatively using albumin, bioimpedance and NRS-2002. A dietitian will also perform a physical examination | *Goal:* Meet requirements estimated using 25-30 kcal/kg IBW per day and 1.5g/kg IBW protein per day  *Intervention:* Control group given no specific advice except recommendation for high energy nutritional supplement drinks on decision of the surgeon. Intervention group provided personalized recommendations from a dietitian to prevent or correct malnutrition. If necessary, patients are prescribed oral nutritional supplements to increase consumption of calories *and* protein  *Reference:* Not specified | To increase compliance patients will be asked to fill in a diary to record their daily prehab practice. Study staff contact patients by telephone weekly to inquire about adherence to the study | *Outcomes measured:* NRS-2002, bioimpedance and albumin  *Timepoint:* Pre-surgery, postoperatively at 3, 6 and 12 months |
| Janssen, 2020 (41)  Cohort ‘before and after’ study | *Cancer type*: Colorectal (77% participants) and AAA (23% participants)  *Sample size*: Prehabilitation group n=267, Control group n=360 | Indication for referral to a dietitian was assessed by the nurse practitioner. A dietitian was consulted if there was undernourishment, decreased appetite, or unintentional weight loss. Nutritional status was assessed using the SNAQ score.  A dietitian assessed nutritional status using the MNA-SF, BMI and blood prealbumin, vitamin B (unspecified) and D. | *Goal:* Meet a minimum daily intake of 1.2g/kg protein per day and caloric intake of patients’ basal metabolic rate plus 30%  *Intervention:* Patients were given dietary advice to ensure they met these requirements. They were given vitamins in the case of depletion and supplemental protein drinks (protein type not specified) when daily protein intake was not sufficient  *Reference:* Not specified | Not specified | No nutrition outcomes measured |
| Moore, 2020 (33)  Description of a prehabilitation program | *Cancer type*: Mixed (Colorectal, lung and esophago-gastric)  *Sample size*: Not applicable | ‘Bespoke nutritional screening’ takes into account BMI, weight trend (loss or gain), and changes in ability to swallow solid food and handgrip strength. | *Goal:* Not stated  *Intervention:* Pathways aimed to incorporate nutrition support to complement existing provision within cancer patient pathways  *Reference:* Not specified | Not specified | *Outcomes measured:* Weight and handgrip strength  *Timepoint:* 4-6 weeks postoperatively and at point of discharge from the service following 12-week recovery program |
| Beck, 2020 (36)  Qualitative study | *Cancer type*: Mixed (Colorectal and ovarian)  *Sample size*: n=16 | No nutritional screening or assessment detailed | *Goal:* Not stated  *Intervention:* Leaflet contained 6 general recommendations including nutrition. Patients were recommended to eat food rich in protein on a daily basis. A range of food choices were listed and 3 recipes for protein shakes were provided  *Reference:* Not specified | Not specified | No nutrition outcomes measured |
| van der Vlies, 2020 (40)  Cohort study | *Cancer type*: Colorectal  *Sample size*: Pre-intervention n=320, Intervention n=146 | Analysis of physical frailty included nutritional status using MNA and weight loss ≥3kg | *Goal:* Not stated  *Intervention:* Referral to a dietitian (n=42, 33.1%) including tube feeding (n=7, 5.5%) and total parenteral nutrition (n=3, 2.4%).  *Reference:* Not specified | Not specified | No nutrition outcomes measured |
| Chmelo, 2020 (20)  Protocol for a feasibility study | *Cancer type*: Mixed (Esophageal and gastric)  *Sample size*: Aims to recruit n=40 | No nutritional screening or assessment detailed | *Goal:* Not stated  *Intervention:* Usual care for all participants will include review by specialist dietitians if they are struggling nutritionally. If unable to manage sufficient calorie input without assistance a naso-enteric feeding tube or feeding jejunostomy may be placed  *Reference:* Not specified | Participants will be contacted once per week by phone to provide support, reinforce program aims and benefits. Activities and exercises will be monitored; however, did not specify if nutrition was monitored | *Outcomes measured:* Sarcopenia by change in amount of L3 skeletal muscle and change in body fat composition both measured by CT and change in handgrip strength  *Timepoint:* Post intervention |
| Sheill, 2020 (21)  Protocol for an RCT | *Cancer type*: Mixed (Esophageal and lung)  *Sample size*: Aims to recruit n=78 (n=39 per group) | Intervention group will receive additional tailored dietetic assessment. Nutritional status determined by dietary interview and body composition by anthropometry | *Goal:* Sessions focus on ensuring adequate dietary energy (25-30kcal/kg) and protein (1.25-1.5g/kg per day) intake  *Intervention:* No detail provided  *Reference:* ESPEN guidelines: Arends J, Bachmann P, Baracos V et al. ESPEN guidelines on nutrition in cancer patients. Clin Nutr. 2017; 36(1):11–48 | Not specified | *Outcomes measured*: Dietary interview and body composition  *Timepoint:* Post intervention, timepoints not specified. |
| Barberan-Garcia, 2020 (22)  Protocol for an RCT | *Cancer type*: Lung  *Sample size*: Aims to recruit n=158 (n=79 per group) | Standard care group- MUST score. Intervention group- not specified, likely standard of care. | *Goal:* Protein intake 1.2-1.5g/kg per day for the intervention group.  *Intervention:* Standard care group- intervention performed by a registered dietitian if MUST ≥2, no other details provided regarding the intervention. Intervention group- personalized dietary counselling by a registered dietitian of a healthy balanced diet or diet adapted to digestive symptoms if present. Protein intake distributed in three main daily meals, by means of food enrichment, and/or nutritional supplementation such as whey protein powder or casein. States that sufficient caloric intake will be ensured, and personalized educational material provided.  *Reference:* Not specified | Standard care group- not specified. Intervention group- follow up surveys using the mobile app. Nutritional personalized education material will also be provided using the mobile app, registered in the technological platform and assessed by the dietitian | *Outcomes measured:* Lean mass index measured by bioimpedanciometry for the intervention group  *Timepoint:* Not specified |
| Ngo-Huang, 2019 (39)  Cohort study | *Cancer type*: Pancreatic  *Sample size*: n=50 | No nutritional screening or assessment detailed | *Goal:* Meet daily calorie and protein goals  *Intervention:* All patients met with a registered dietitian. Provided with individualized nutrition recommendations including, but not limited to, meeting estimated requirements and strategies for management of treatment or disease-related side effects. All patients were encouraged to consume a high protein meal, snack or supplement (not specified) within an hour of completion of strengthening exercises  *Reference:* Not specified | Not specified | No nutrition outcomes measured |
| Liu, 2020 (48)  RCT | *Cancer type*: Lung  *Sample size*: Prehabilitation group n=37, Control group n=36 | All participants completed a 3-day total food recall questionnaire to assess daily caloric and protein intake | *Goal:* To achieve adequate protein intake of 1.5g/kg per day for the intervention group  *Intervention:* All participants were advised to change unhealthy eating habits, avoid high calorie and high fat diets, eat more fruit and vegetables and ingest more high-quality protein. Intervention group received whey protein powder to consume daily within 1 hour of exercise.  *Reference:* McClave SA, Kozar R, Martindale RG, et al. Summary points and consensus recommendations from the North American surgical nutrition summit. JPEN J Parenter Enteral Nutr. 2013;37:99S–105S | Not specified | No nutrition outcomes measured |
| Minnella, 2019 (49)  RCT | *Cancer type*: Bladder  *Sample size*: Prehabilitation group n=35, Control group n=35 | Using a self-reported 3-day food record a registered dietitian performed a baseline evaluation and estimated individual energy and macronutrient requirements | *Goal:* Aimed to guarantee adequate caloric and macronutrient content and to avoid both under and over feeding. Harris-Benedict equation estimated caloric requirements using 1.2 stress factor and daily protein aimed at 1.5g/kg per day IBW.  *Intervention:* If needed, a whey protein supplement was prescribed to achieve this goal. Participants were educated to consume this after exercise on training days.  *Reference:* Weimann A, Braga M, Carli F et al. [ESPEN guideline: clinical nutrition in surgery](https://www.sciencedirect.com/science/article/pii/S0261561417300638) Clin Nutr. 2017; 36:623-650 | Weekly telephone call to assess adherence to both training and nutrition protocols and protein sachet consumed. During the phone call further clarification on nutrition was provided to the participants if needed | No nutrition outcomes measured |
| van Rooijen, 2019 (35)  Feasibility | *Cancer type*: Colorectal  *Sample size*: Prehabilitation group n=20, Control group n=30 | PG-SGA, skin fold measurements, anthropometry, BMI and 3-day food diary | *Goal:* Intervention group aimed total protein intake 1.5-1.8g/kg protein per day  *Intervention:* Intervention group received dietary advice, vitamin D and multivitamins (50% RDA) and protein supplements. Protein supplements (Refit TMP 90 shake) were 0.4g/kg of body weight per serving and given twice a day during the program with one portion being within 1 hour of exercise and the other before bedtime  *Reference:* Morton RW, Murphy KT, McKellar SR, et al: A systematic review, meta-analysis and meta-regression of the effect of protein supplementation on resistance training-induced gains in muscle mass and strength in healthy adults. Br J Sports Med 2018;52:376–84  Deutz NE, Bauer JM, Barazzoni R, et al: Protein intake and exercise for optimal muscle function with aging: recommendations from the ESPEN Expert Group. Clin Nutr 2014;33:929–36 | Patients were phoned weekly by a specialist nurse to increase adherence to the program | Height, weight, % weight loss, handgrip strength and PG-SGA at 30-day postoperative follow-up but were too few to perform proper analysis |
| Janssen, 2019 (23)  Protocol for a ‘before and after’ study | *Cancer type*: Colorectal and AAA (aiming 1:1 recruitment)  *Sample size*: Aims to recruit n=275 | BMI, MNA-SF, SNAQ score and blood levels of folic acid, vitamin B (unspecified) and D, lipid profile and prealbumin | *Goal:* Protein 1.2g/kg body weight (BMI<30) per day and calories based on WHO formula for basal need plus 30%  *Intervention:* As per hospitals’ protocol malnourished patients (MNA-SF <12) are provided dietary advice on required protein and calorie intake. Supplements (not specified) were provided when required protein and calorie intake was still not met after dietary advice.  *Reference:* Author stated WHO formula used calorie requirements; protein not referenced | Not specified | No nutrition outcomes measured |
| Dewberry, 2019 (30)  Pilot study | *Cancer type*: Esophageal  *Sample size*: Prehabilitation group n=11, Control group n=11 | ASPEN malnutrition assessment tool and physical assessment for muscle loss | *Goal:* Optimize preoperative nutritional status  *Intervention:* The cancer center dietitian had weekly meetings with patients that consisted of diet recall and counseling on side effect management  *Reference:* Not specified | Patient compliance with nutritional guidance was assessed through measurement of weight maintenance | *Outcomes measured:* Percentage weight loss and albumin  *Timepoint:* Not specified |
| van Rooijen, 2019 (27)  International RCT protocol | *Cancer type*: Colorectal  *Sample size*: Aims to recruit total n=714 (n=357 per group) | PG-SGA, body composition (skinfold measurements, mid-upper arm muscle area and handgrip strength) and nutritional intake by 3-day food diary | *Goal:* Target protein intake 1.5-1.8g/kg body weight per day. In cachectic and sarcopenic patients aim to increase lean body mass by 1-2kg or more during the 4 weeks of prehabilitation  *Intervention:* Participants will receive high quality protein supplements containing 30g whey protein following exercise and before sleep. Dietary advice given to achieve oral protein intake spread evenly across meals. Vitamin D supplemented daily (10 μg for women aged 50-69y, for men <70y and women <50y with coloured skin and/or little sun exposure and 20 μg for women and men aged 70 y or older) and all other vitamins and minerals supplied in a multivitamin/mineral supplement containing 50% of the recommended daily allowance.  *Reference:* Cermak NM, Res PT, De Groot LC, et al. Protein supplementation augments the adaptive response of skeletal muscle to resistance-type exercise training: a meta-analysis. Am J Clin Nutr. 2012;96(6).  Gillis C, Loiselle SE, Fiore JF et al. Prehabilitation with whey protein supplementation on perioperative functional exercise capacity in patients undergoing colorectal resection for Cancer: a pilot double-blinded randomized placebo-controlled trial. J Acad Nutr Diet. 2016;116(5). | Not specified | *Outcomes measured:* 3-day food diary to calculate caloric and protein intake and PG-SGA  *Timepoint*: One week before surgery, 4 weeks, 8 weeks and 1 year postoperatively |
| Allen, 2018 (24)  Protocol for an RCT | *Cancer type*: Mixed (Esophageal and gastric)  *Sample size*: Aims to recruit n=48 (n=24 per group) | Handgrip strength, mid-arm muscle circumference, triceps skinfold thickness and sarcopenia. | *Goal:* Not stated  *Intervention:* All patients will receive frequent, tailored dietetic input, with calorie and protein intake increases where appropriate.  *Reference:* Not specified | Frequent tailored dietetic input with regular telephone calls consultations | *Outcomes measured:* Weight, handgrip strength, mid-arm muscle circumference and triceps skinfold thickness  *Timepoint:* 2 weeks, 6 weeks and 6 months following hospital discharge |
| Bruns, 2019 (28)  Pilot study | *Cancer type*: Colorectal  *Sample size*: n=14 | MNA-SF, weight loss (>4.5kg in the past year), handgrip strength (sex and BMI specific cutoff points used to determine low grip strength) | *Goal:* Protein 1.5-1.8g/kg per day  *Intervention:* 7-day menu consisting of two small meals per day (breakfast and snack 20-30g protein in each). To prepare the meals, the ingredients were delivered at home before the start of the program.  *Reference:* Author stated ESPEN guidelines used for protein intake | One week after initiation of the program the patient was contacted by phone to investigate any issues or questions. Compliance was assessed during the phone call and at the end of the program by asking how many days during the previous week they had followed the recipes | No nutrition outcomes measured |
| Nakajima, 2019 (38)  Cohort study | *Cancer type*: Mixed (Hepatic and pancreatic)  *Sample size*: Prehabilitation group n=76, Control group n=76 | Body weight, BMI, albumin and prognostic nutritional index | *Goal:* Not stated  *Intervention:* Patients were asked to take a leucine-rich essential amino acid supplement within 30 min after the start and end of exercise therapy  *Reference:* Not specified | Not specified | *Outcomes measured:* Body weight, BMI, albumin and prognostic nutritional index  *Timepoint:* At time of surgery |
| Souwer, 2018 (37)  Cohort study | *Cancer type*: Colorectal  *Sample size*: Prehabilitation group n=86, During development of prehabilitation n=75, Control group (prior to prehabilitation) n=63 | All patients were referred to a dietitian for a full nutritional assessment. SNAQ questionnaire was performed for all patients. | *Goal:* Protein 1.2-1.5g/kg per day  *Intervention:* Subsequent nutrition support to target this daily protein intake  *Reference:* Wolfe RR, Miller SL, Miller KB. [Optimal protein intake in the elderly](https://www.sciencedirect.com/science/article/pii/S0261561408001179). Clin Nutr. 2008; 27: 675-684  Jeevanandam M, Lowry SF, Horowitz GD et al; [Influence of increasing dietary intake on whole body protein kinetics in normal man](https://www.sciencedirect.com/science/article/pii/0261561486900415). Clin Nutr. 1986; 5:41-48 | Not specified | No nutrition outcomes measured |
| Minnella, 2018 (50)  RCT | *Cancer type*: Mixed (Esophageal and gastric)  *Sample size*: Prehabilitation group n=26, Control group n=25 | 3-day food record (2 weekdays, 1 weekend). A dietitian assessed dietary habits, anthropometric data, and estimated the required amount and relative proportion of macronutrients, to create a comprehensive status evaluation. | *Goal:* Protein 1.2-1.5g/kg per day IBW or approximately 20% of total energy requirements  *Intervention:* Food-based dietary advice was given and a whey protein supplement to guarantee daily protein intake above. These supplements (if needed) were consumed every morning after breakfast or immediately after exercise during training days. Nutrition therapy was given to all participants in the intervention group, even in the absence of malnutrition  *Reference:* Braga M, Ljungqvist O, Soeters P et al. ESPEN. ESPEN guidelines on parenteral nutrition: surgery. Clin Nutr. 2009;28 (4):378-386. | Participants were provided with a logbook, and the nutritionist monitored the adherence and addressed issues or doubts by weekly telephone calls | No nutrition outcomes measured |
| McIsaac, 2018 (26)  Protocol for an RCT | *Cancer type*: Mixed (Colorectal, thoracic, hepatobiliary and urological)  *Sample size*: Aims to recruit n=200 (n=100 per group) | No nutritional screening or assessment detailed | *Goal:* Not stated  *Intervention:* Control group will receive Canada’s food guide. Intervention group also provided with standard nutritional advice  *Reference:* Not specified | Activity logs and weekly phone calls will be used to encourage and measure compliance and to answer questions. Does not specify if nutrition will be monitored | No nutrition outcomes measured |
| Paterson, 2019 (43)  Cohort study | *Cancer type*: Prostate  *Sample size*: n=34 | No nutritional screening or assessment detailed | *Goal:* Not stated  *Intervention:* Participants were provided with a custom-made evidence-based self-management booklet and attended a seminar, both of which included nutrition as a topic.  *Reference:* Not specified | Not specified | No nutrition outcomes measured |
| Macleod, 2018 (44)  Cohort study | *Cancer type*: Colorectal  *Sample size*: n=22 | BMI in intervention group | *Goal:* No goal was set during prehabilitation intervention.  *Intervention:* During prehabilitation, participants not at risk of malnutrition (BMI>20kg/m^2^) focused on avoiding weight gain and increasing nutrient quality of their diet in line with Department of Health Eatwell guide.  *Reference:* Not specified | Three face to face contacts (once at start of prehabilitation and another two postoperatively) and a minimum of nine phone calls were undertaken. Did not specify if nutrition was monitored | *Outcomes measured:* Weight, BMI, waist circumference and dietary intake including fat and fibre intake using the Dietary Instrument for Nutrition Education questionnaire  *Timepoint:* Not specified |
| Bousquet-Dion, 2018 (51)  RCT | *Cancer type*: Colorectal  *Sample size*: Prehabilitation group n=41, Control group n=39 | All participants had their nutritional status assessed by a registered dietitian. Nutritional status was evaluated using PG-SGA, NRS-2002 and 3-day food diary from which carbohydrate, fat and protein quantities were estimated using food exchange lists and composition tables. Macronutrient intake was evaluated based on Dietary Reference Intake Values and food choices were compared to Eating Well with Canada’s Food Guide recommendations. | *Goal:* Protein 1.2g/kg per day body weight (or adjusted body weight in obese patients)  *Intervention:* As part of routine care if the patient did not meet the protein requirement by diet alone, they were provided with whey protein supplementation to meet this goal. Patients were instructed to ingest protein and/or the supplements within one hour of their exercise training. Further nutritional counselling was given to help with bowel movements regularity, body composition optimization and glycemic control.  *Reference:* Weimann A, Braga M, Harsanyi L, et al. ESPEN guidelines on enteral nutrition: surgery including organ transplantation. Clin Nutr. 2006;25:224–244 | Patients were contacted on a weekly basis by telephone and were asked a standardized set of questions. Did not specify if nutrition was monitored | *Outcomes measures:* Bioelectrical impedance analysis to determine weight, body fat percentage and lean body mass  *Timepoint*:4 and 8 weeks postoperatively |
| Ngo-Huang, 2017 (45)  Cohort study | *Cancer type*: Pancreatic  *Sample size*: n=20 | No nutritional screening or assessment detailed | *Goal:* Not stated  *Intervention:* A clinical dietitian provided instruction to consume a high-protein meal or snack (at least 20 g of protein) within 1 h after performing strengthening exercises and provided guidance on appropriate food selection.  *Reference:* Not specified | Research staff called patients every 2 weeks to encourage adherence and screen for nutrition-related issues. | No nutrition outcomes measured |
| Mazzola, 2017 (46)  Cohort study | *Cancer type*: Mixed (Esophageal, gastric and pancreatic)  *Sample size*: Prehabilitation group n=41, Control group n=35 | Weight loss, BMI, MUST and albumin. High nutritional risk was defined as weight loss >10-15% in the last 6 months, BMI <18.5kg/m^2^ or MUST >2 | *Goal:* Not stated  *Intervention*: High nutritional risk patients received oral nutritional support (Impact Oral, 2 “bricks” during 12 h) for 5-7 days prior to surgery. In the majority of patients nutritional support was performed by administering a mixture containing immuno-nutritional products. Where patients were not able to take oral nutritional support, an admission to the Clinic’s Nutrition Unit was scheduled for adequate nutritional implementation by means of naso-jejunal feeding tube, while only conditions of particular clinical complexity required parenteral nutrition  *Reference:* Not specified | Not specified | No nutrition outcomes measured |
| Le Roy, 2016 (25)  Protocol for an RCT | *Cancer type*: Mixed (Esophageal and gastric)  *Sample size*: Aims to recruit total n=180 (n=60 per group) | A nutritionist will perform a medical examination running appropriate biological tests to evaluate the nutritional status | *Goal:* Protein 1.2g/kg body weight per day (adjusted body weight used for obese patients)  *Intervention:* Individualized care plan provided to each patient. Patients will be asked to consume a protein supplement (not specified) within 1 hour of their exercise regime  *Reference:* Weimann A, Braga M, Harsanyi L, et al. ESPEN Guidelines on Enteral Nutrition: Surgery including organ transplantation. Clin Nutr 2006;25:224–44 | A dietitian will assess compliance of nutritional support at each cycle of chemotherapy and will adjust it if necessary | No nutrition outcomes measured |
| Jensen, 2016 (52)  RCT | *Cancer type*: Bladder  *Sample size*: Prehabilitation group n=50, Control group n=57 | All patients were screened for nutritional status using the NRS-2002 | *Goal:* Not stated  *Intervention:* As part of routine care all patients were offered oral supplements (not specified) and average protein and calorie intake was calculated. Intervention group also received dietary instruction to increase protein intake  *Reference:* Not specified | All patients were instructed to monitor daily food intake for 2 weeks by using a food log. | No nutrition outcomes measured |
| Huang, 2016 (47)  Cohort study | *Cancer type*: Mixed (Esophageal, gastric, colorectal and thoracic surgery)  *Sample size*: n=26 | No nutritional screening or assessment detailed | *Goal:* Not stated  *Intervention:* Referred for nutritional input prior to surgery at the discretion of the anesthetist if clinically warranted  *Reference:* Not specified | Not specified | No nutrition outcomes measured |
| Gillis, 2016 (8)  Pilot study | *Cancer type*: Colorectal  *Sample size*: Prehabilitation group n=21, Control group n=22 | 3-day food diary (2 weekdays and 1 weekend) recording the quantity of all foods and beverages consumed using standard household measurements. PG-SGA and NRS-2002 as well as biochemical, functional and anthropometric measurements. Dietary intake was determined through analysis of food records and evaluated based on individually calculated requirements as above and food choices were compared with Canada’s Food Guide recommendations. | *Goal:* Individual protein needs calculated as 20% total energy expenditure (approx 1.2-1.5 g protein/kg per day), determined by using indirect calorimetry  *Intervention:* Both groups participated in identical counselling sessions (90 minutes) and provided with personalized nutrition care plans focused on meeting requirements with appropriate food choices, management of cancer-related symptoms, blood glucose control, optimization of body composition (weight loss or gain if necessary), and nutrient intake by using practical suggestions. Patients in the intervention arm were instructed to take a whey protein supplement (in a quantity that matched the patient’s need according to the estimated deficit in dietary protein intake) once per day for 4 weeks preoperatively and 4 weeks postoperatively.  *Reference:* Braga M, Ljungqvist O, Soeters P et al. ESPEN guidelines on parenteral nutrition: surgery. Clin Nutr. 2009;28 (4):378-386. | Compliance was measured by using a diary to document the quantity of the nutritional supplement taken each day. Patients were contacted weekly and queried with a standardized set of open-ended questions designed to identify problems with compliance to the supplement regimen. | *Outcomes measured:* Change in lean and fat mass measured using bioelectrical impedance analysis, change in handgrip strength  *Timepoint:* 4 weeks postoperatively |
| Santa Mina, 2014 (29)  Pilot RCT | *Cancer type*: Prostate  *Sample size*: Aims to recruit n=100 (n=50 per group) | Height, weight, BMI, body composition by bioelectrical impedance, waist circumference and handgrip strength recorded but not stated to be part of a nutrition assessment. | *Goal*: Not stated  *Intervention:* Both groups received a copy of a lifestyle support group which contained information on nutrition  *Reference:* Not specified | Not specified | Outcomes measured: Waist circumference, BMI and body fat percentage measured through bioelectrical impedance  Timepoint: 1 week prior to surgery and 4, 12 and 26 weeks postoperatively |
| Gillis, 2014 (53)  RCT | *Cancer type*: Colorectal  *Sample size*: Prehabilitation group n=38, Control group n=39 | 3 day estimated food record (2 weekdays and 1 weekend) measuring the quantity of all foods and beverages consumed and methods of preparation which were assessed by a dietitian. Medical exam, patient questionnaires, biochemical, functional and anthropometric measurements considered in the assessment. Macronutrient quantities (grams of carbohydrate, fat, and protein consumed) were estimated from each patient’s food record with food exchange lists and composition tables and evaluated based on Dietary Reference Intake values. Food choices were compared to Eating Well with Canada’s Food Guide recommendations. | *Goal:* Individual protein requirements were calculated as 1.2g of protein per kilogram of body weight per day (adjusted body weight was used for obese patients)  *Intervention:* A dietitian provided individualized care to meet estimated needs based on the 3-day food diary. All patients were given a whey protein supplement to guarantee adequate daily protein intake at a quantity that matched the estimated dietary deficit. Patients were asked to consume the protein supplement within 1h of their exercise regimen. Recipes to improve the palatability of the product were also provided.  Nutritional care plans then focused on management of cancer-related symptoms, blood glucose control if necessary, optimization of body composition (weight loss/gain if necessary), and appropriate balance of food choices by providing practical suggestions based on actual intake.  *Reference:* Weimann A, Braga M, Harsanyi et al ESPEN Guidelines on Enteral Nutrition: Surgery including organ transplantation. Clin Nutr 2006; 25:224–44 | To facilitate adherence to the program all patients received a standard instructional booklet describing all elements of the program in detail. The booklet also contained a diary where the patients were asked to document all activities related to the program.  To encourage and measure adherence, patients were contacted weekly by telephone and assessed with a standardized set of open-ended questions to uncover issues related to maintaining compliance to the amount of whey protein ingested. | No nutrition outcomes measured |
| Li, 2013 (31)  Pilot study | *Cancer type*: Colorectal  *Sample size*: Prehabilitation group n=42, Control group n=45 | Patients enrolled in the prehabilitation program were evaluated by a nutritionist during a 1-hour visit and with an SGA. | *Goal:* Protein 1.2 g/kg body weight per day  *Intervention:* One or two modifiable dietary behaviors were identified and discussed with the patient. The patients were provided with whey protein isolate which was used as a nutritious food supplement to guarantee a daily intake of protein as per goal listed above. Recipes to make intake palatable were given. Patients were asked to consume protein preferably within 1 h of their exercise regimen  *Reference:* Not specified | Not specified | No nutrition outcomes measured |
| Carli, 2012 (34)  Case report | *Cancer type*: Ovarian  *Sample size*: n=1 | Nutritional assessment using albumin and standard 24-hour dietary recall. | *Goal:* Not stated  *Intervention:* The patient was encouraged to increase her dietary intake of protein and calories, including a 30 g daily supplement of soy kefir per day  *Reference:* Not specified | Not specified | *Outcome measures:* A ‘nutritional evaluation’ conducted, but not reported  *Timepoint:* 4 and 8 weeks postoperatively |

BMI (Body mass index); NRS-2002 (Nutritional risk score-2002 questionnaire); IBW (Ideal body weight); SNAQ (Short nutritional assessment questionnaire); MNA-SF (Mini nutritional assessment short form); MUST (Malnutrition universal screening tool); PG-SGA (Patient generated subjective global assessment); SGA (Subjective global assessment); AAA (Abdominal aortic aneurysm).
